# Association between immune-related adverse events and efficacy of PD-1 inhibitors in Chinese patients with advanced melanoma

**DOI:** 10.18632/aging.103285

**Published:** 2020-06-08

**Authors:** Jing-Jing Zhao, Xi-Zhi Wen, Ya Ding, Dan-Dan Li, Bao-Yan Zhu, Jing-Jing Li, De-Sheng Weng, Xing Zhang, Xiao-Shi Zhang

**Affiliations:** 1 Sun Yat-Sen University Cancer Center, State Key Laboratory of Oncology in South China, Collaborative Innovation Center for Cancer Medicine, Guangzhou, China; 2 Department of Biotherapy, Sun Yat-Sen University Cancer Center, Guangzhou, China

**Keywords:** immune-related adverse events, PD-1 inhibitor, immune checkpoint inhibitors, advanced melanoma, Chinese patients

## Abstract

Programmed cell death 1 (PD-1) checkpoint inhibitor therapy leads to immune-related adverse events (irAEs). We sought to evaluate whether the development of irAEs correlates with the treatment response in Chinese patients with advanced melanoma. In this study, we conducted a retrospective study of advanced melanoma patients who received PD-1 inhibitor therapy in China between August 2014 and March 2018. A total of 93 patients treated with PD-1 inhibitors including pembrolizumab and nivolumab were enrolled. The most frequent irAEs were pruritus, rash, vitiligo, and fatigue. The median time to onset of irAEs was 6.1 weeks. The overall response rate (ORR) and disease control rate (DCR) were higher in patients with irAEs than those without irAEs (*P* = 0.004 and *P* = 0.003, respectively), and better in patients who experienced three or more irAEs than those with none (*P* <0.001 and *P* <0.001, respectively). The ORR and DCR were significantly better in patients with grade 1 to 2 irAEs when compared with those with none (*P* = 0.002 and *P* = 0.003, respectively). In addition, the median progression-free survival and overall survival were longer in patients with irAEs than in those without irAEs (*P* = 0.007 and *P* = 0.002, respectively). In conclusion, our data demonstrated that irAEs were associated with a better clinical outcome after treatment with PD-1 inhibitor therapy in Chinese patients with advanced melanoma.

## INTRODUCTION

Malignant melanoma is the leading cause of cancer-related deaths worldwide, and its incidence in China has been increasing in the recent years [1, 2]. The treatment of advanced melanoma has remarkably improved due to the successful clinical development of immune checkpoint inhibitors (ICIs) that reactivate the anticancer immune response [3, 4]. The anti-programmed cell death 1 (PD-1) receptor antibody represents the second breakthrough in immune checkpoint blockade therapy of melanoma after the approval of ipilimumab [5–8]. Two anti-PD-1 antibodies, pembrolizumab, and nivolumab were approved for the treatment of melanoma in 2014 [6, 9].

PD-1 is a key inhibitory receptor expressed on activated CD4^+^ T cells, CD8^+^ T cells, natural killer T cells, and B cells [10–14]. Its binding with programmed cell death ligands (PD-L1 and PD-L2) expressed on antigen-presenting cells, and human cancers including melanoma delivers a negative signal to lymphocytes that inhibits T-cell proliferation, cytokine release, and cytotoxicity [10–14]. Anti-PD-1 antibodies including pembrolizumab and nivolumab can reverse this T-cell suppression and induce long-lasting antitumor responses in patients with advanced solid tumors, including advanced melanoma [15–17]. By activating the immune system, the anti-PD-1 antibodies lead to autoimmune-like toxicities known as immune-related adverse events (irAEs) through immune cell infiltration into normal, noncancerous tissues [6]. Such irAEs were not frequently observed with cytotoxic chemotherapy or other classes of targeted agents [5, 18]. The irAEs in response to ICIs have varying times to onset and include organ-specific toxicities in skin, endocrine, gastrointestinal, hepatobiliary, pulmonary, and renal, as well as non-organ-specific toxicities such as fatigue, pyrexia, appetite loss, arthralgia, and myalgia [19–22]. Their presentation can range from mild and manageable, to severe and life threatening if not recognized early and treated with appropriate measures such as corticosteroids [5, 23].

Recent studies have demonstrated that the development of irAEs in melanoma [11, 24–27] and non–small cell lung cancer (NSCLC) [12, 18, 20, 28, 29] patients treated with ICIs could correlate with the clinical response. Chinese melanoma patients have a higher proportion of the acral and mucosal types, which have distinct genetic and clinical characteristics, lower somatic mutational burden, and poorer prognoses [30–33]. However, the relationship between the irAEs and clinical outcomes remains unclear. In this study, we performed a retrospective analysis of the clinical data obtained from 93 advanced melanoma patients treated with anti-PD-1 antibodies at the Sun Yat-sen University Cancer Center and evaluated the association between irAEs and clinical outcomes. The results of this study will help identify patients with advanced melanoma who are most likely to benefit from PD-1 checkpoint inhibitor therapy.

## RESULTS

### Patient characteristics

As list in Table 1, a total of 93 patients with advanced melanoma treated with PD-1 inhibitors at our center between August 2014 and March 2018 were enrolled in this study. Of these patients, 59 (63.4%) and 34 (36.6%) were treated with pembrolizumab and nivolumab, respectively. The median number of doses of anti-PD-1 inhibitors doses was 5 (range, 2–24). The cohort comprised of 54 men (58.1%) and 39 women (41.9%), with a median age of 52 years (range, 22-78 years). The primary lesions were acral melanomas that arose from palms, soles, and subungual sites in 26 patients (28.0%), chronic sun-derived (CSD) or non-CSD melanomas that arose in non-acral sites in 34 patients (36.5%), mucosal melanomas in 21 patients (22.6%) and uveal in 3 patients (3.2%). Nine patients (9.7%) had no primary lesions. While 33 patients (35.5%) had elevated serum LDH levels, 21 (22.6%) harbored BRAF V600E mutation and 2 (2.2%) harbored C-KIT mutation. Ten patients (10.8%) had brain metastases, and they received radiotherapy for the brain lesions before the infusion of anti-PD-1 antibodies. A total of 61 patients (65.6%) had received prior ipilimumab, chemotherapy or BRAF inhibitors. Overall, irAEs were noted in 54 patients (58.1%). No significant differences were observed in the baseline characteristics of the patients with and without irAEs (Table 1, *P* > 0.05).

**Table 1:** Distribution of demographic and clinical characteristics of patients.

**Characteristics**	**Patients no. (%)**			
	**Total (n=93)**	**With irAEs (n=54)**	**Without irAEs (n=39)**	***P***
**Age, mean(range)**	52(22-78)	52(25-77)	53(22-78)	0.827
**Gender**				0.784
male	54(58.1)	32(59.3)	22(56.4)	
female	39(41.9)	22(40.7)	17(43.6)	
**ECOG status**				0.207
0-1	84(90.3)	47(87.0)	37(94.9)	
≥2	9(9.7)	7(13.0)	2(5.1)	
**Primary site**				0.653
Acral	26(28.0)	18(33.3)	8(20.5)	
CSD/non-CSD	34(36.5)	19(35.2)	15(38.5)	
Mucosal	21(22.6)	10(18.5)	11(28.2)	
Uveal	3(3.2)	2(3.7)	1(2.6)	
No-primary lesion	9(9.7)	5(9.3)	4(10.2)	
**Metastasis stage ^a^**				0.719
M1a	24(25.8)	15(27.8)	9(23.1)	
M1b	20(21.5)	13(24.1)	7(17.9)	
M1c	39(41.9)	20(37.0)	19(48.7)	
M1d	10(10.8)	6(11.1)	4(10.3)	
**LDH level**				0.944
≤UNL	60(64.5)	35(64.8)	25(64.1)	
>UNL	33(35.5)	19(35.2)	14(35.9)	
**Brain metastasis**				0.896
Yes	10(10.8)	6(11.1)	4(10.3)	
No	83(89.2)	48(88.9)	35(89.7)	
**Liver metastasis**				0.484
Yes	32(34.4)	17(31.5)	15(38.5)	
No	61(65.6)	37(68.5)	24(61.5)	
**Lung metastasis**				0.978
Yes	55(59.1)	32(59.3)	23(59.0)	
No	38(40.9)	22(40.7)	16(41.0)	
**BRAF ^V600E^ status**				0.270
mutation	21(22.6)	10(18.5)	11(28.2)	
wild-type	72(77.4)	44(81.5)	28(71.8)	
**C-KIT status**				0.093
mutation	2	0(0)	2(5.1%)	
wild-type	91	54(100%)	37(94.9%)	
**Prior therapy ^b^**				0.530
Yes	61(65.6)	34(63.0)	27(69.2)	
No	32(34.4)	20(37.0)	12(30.8)	
**PD-1 inhibitor**				0.324
Pembrolizumab	59(63.4)	32(59.3)	27(69.2)	
Nivolumab	34(36.6)	22(40.7)	12(30.8)	

Abbreviations: ECOG, Eastern Cooperative Oncology Group; CSD, chronic sun derived; LDH, Lactate dehydrogenase; ULN, upper limit of normal

^a^ According to the 7^th^ edition of the AJCC staging manual.

^b^ Including ipilimumab, chemotherapy or BRAF inhibitors for patients with BRAF mutation.

### IrAEs profile of the patients

IrAEs occurred in 58.1% of the patients (54/93), which were grade 1-2 in 46 patients (49.5%) and grade 3-4 in 8 (8.6%) patients (Table 2). The observed irAEs were skin (67.8%), endocrine (11.8%), gastrointestinal (6.5%), hepatobiliary (17.2%), and others (25.8%) (Table 2). No pulmonary and renal events were reported in this study population. The most common skin irAEs were pruritus (30.1%), rash (24.7%), and vitiligo (16.1%), and the most common endocrine irAEs were thyroiditis/hypothyroidism (7.5%) and hypoadrenocorticism (6.5%). Hepatitis (8.6%) and elevated transaminase levels (7.5%) were the most common hepatobiliary irAEs observed (Table 2). The other irAEs included fatigue (15.1%) and pyrexia (8.6%). Steroids were used to treat the irAEs in 8 patients (8.6%) (Table 2). Median time to onset in weeks was 7.4 (range, 0.1-36.3), 11.9 (1.0-36.4), 4.6 (0.4-21.7), 6.6 (1.0-24.7) and 3.9 (0.1-18.6) for skin, endocrine, gastrointestinal, hepatobiliary and other irAEs, respectively, of any grade (Table 2).

**Table 2:** Immune-related adverse events according to category and grade.

**Category**	**Patients no. (%)**				**Weeks to Onset, Median (range)**
	**Total (n=93)**	**Grade 1-2**	**Grade 3-4**	**Systemic Steroid Therapy**	
**Any**	54(58.1)	46(49.5)	8(8.6)	8(8.6)	6.1(0.1-36.4)
Skin	40(67.8)				7.4(0.1-36.3)
Rash	23(24.7)	23(24.7)	NA	NA	
Pruritus	28(30.1)	28(30.1)	NA	NA	
Vitiligo	15(16.1)	15(16.1)	NA	NA	
Psoriasis	1(1.1)	1(1.1)	NA	NA	
**Endocrine**	11(11.8)				11.9(1.0-36.4)
Thyroiditis/hypothyroidism	7(7.5)	7(7.5)	NA	NA	
Hypophysitis	1(1.1)	1(1.1)	NA	NA	
Hypoadrenocorticism	6(6.5)	6(6.5)	NA	NA	
Gastrointestinal	6(6.5)				4.6(0.4-21.7)
Diarrhea	5(5.4)	5(5.4)	NA	NA	
Nausea/vomiting	1(1.1)	1(1.1)	NA	NA	
colitis	NA	NA	NA	NA	
**Hepatobiliary**	16(17.2)				6.6(1.0-24.7)
Elevated transaminase	7(7.5)	7(7.5)	NA	NA	
Hyperbilirubinemia	1(1.1)	1(1.1)	NA	NA	
Hepatitis	8(8.6)	3(3.2)	5(5.4)	7(7.5)	
Cholangitis	NA	NA	NA	NA	
**Pulmonary**	NA	NA	NA	NA	
**Renal**	NA	NA	NA	NA	
**Other**	24(25.8)				3.9(0.1-18.6)
Fatigue	14(15.1)	14(15.1)	NA	NA	
Appetite loss	3(3.2)	3(3.2)	NA	NA	
Arthralgia/myalgia	3(3.2)	3(3.2)	NA	NA	
Pyrexia	8(8.6)	5(5.4)	3(3.2)	NA	
Rhabdomyolysis	1(1.1)	NA	1(1.1)	1(1.1)	
Uveitis	1(1.1)	NA	1(1.1)	1(1.1)	

Abbreviations: NA, not applicable.

### Association between irAEs and response rates

In this study, the objective response rate (ORR) and disease control rate (DCR) in patients who received PD-1 inhibitors were 22.6% and 40.9%, respectively (Table 3). The ORR and DCR were significantly better in patients who experienced irAEs than those who did not (33.3% versus 7.7%; *P* = 0.004 and 53.7% versus 23.1%; *P* = 0.003, respectively) (Table 3). The ORR and DCR were a little higher in patients who experienced one to two irAEs than those with no irAEs (19.4% versus 7.7% and 35.5% versus 23.1%, respectively), although the results were not statistically significant (*P* = 0.148 and *P* = 0.254, respectively) (Table 3). Moreover, ORR and DCR were significantly better in patients who experienced three or more irAEs than those who experienced no irAEs (ORR: 42.2% versus 7.7%; *P* < 0.001 and DCR: 78.3% versus 23.1%; *P* < 0.001) and one to two irAEs (ORR: 42.2% versus 19.4%; *P* < 0.001 and DCR: 78.3% versus 35.5% *P* < 0.001) (Table 3). In addition, patients with grade 1 to 2 irAEs had significantly higher ORR and DCR than those with no irAEs (40.0% versus 7.7%; *P* = 0.002 and 54.3% versus 23.1%; *P* = 0.003, respectively) (Table 3). In contrast, no significant difference was found in the ORR and DCR in patients with grade 3 to 4 irAEs when compared with those with no irAEs (12.5% versus 7.7%; *P* = 0.657 and 50.0% versus 23.1%; *P* = 0.121, respectively) (Table 3). In addition, the clinical outcomes in patients with grade 3 to 4 irAEs were poorer when compared with those in patients with grade 1 to 2 irAEs (ORR: 12.5% versus 40%; *P* = 0.176 and DCR: 50.0% versus 54.3%; *P* = 0.820, respectively) (Table 3).

**Table 3:** Impact of immune-related adverse events on response to PD-1 inhibitors therapy.

	**Total (n=93)**	**Number of irAEs**				**irAEs grade**	
		**Any (n=54)**	**None (n=39)**	**1-2 (n=31)**	**≥3 (n=23)**	**1-2 (n=46)**	**3-4 (n=8)**
CR, n (%)	2(2.2)	2(3.7)	0(0.0)	0(0.0)	2(8.7)	2(4.3)	0(0.0)
PR, n (%)	19(20.4)	16(29.6)	3(7.7)	6(19.4)	10(43.5)	15(32.6)	1(12.5)
SD, n (%)	17(18.3)	11(20.4)	6(15.4)	5(16.1)	6(26.1)	8(17.4)	3(37.5)
PD, n (%)	55(59.1)	25(46.3)	30(76.9)	20(64.5)	5(21.7)	21(45.7)	4(50.0)
ORR, % (95% CI)	22.6 (14.0-32.3)	33.3 (20.4-46.3)	7.7 (0.0-17.9)	19.4 (6.5-35.5)	42.2 (30.4-69.6)	40.0 (23.9-50.0)	12.5 (0.0-37.5)
*P_1_*		0.004^a^		0.148^a^	<0.001^a^	0.002^a^	0.657^a^ 0.176^c^
DCR, % (95% CI)	40.9 (31.2-51.6)	53.7 (38.9-66.7)	23.1 (10.3-38.5)	35.5 (19.4-51.6)	78.3 (60.9-95.7)	54.3 (41.3-69.6)	50.0 (12.5-87.5)
*P_2_*		0.003^a^		0.254^a^	<0.001^a^ <0.001^b^	0.003^a^	0.121^a^ 0.820^c^

Abbreviations: irAEs, immune-related adverse events; CR, complete remission; PR, partial remission; SD, stable disease; PD, progressive disease; ORR, objective response rate; DCR, Disease control rate

^a^ Versus no immune-related adverse events.

^b^ Versus 1-2 immune-related adverse events.

^c^ Versus Grade 1-2 immune-related adverse events.

### Association between irAEs and survival

**Figure 1:**
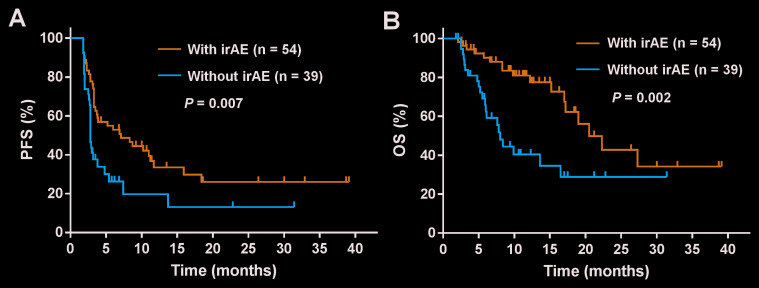
**Kaplan-Meier analysis of survival among patients who experienced an immune-related adverse events (irAEs) or not.** Shown are the curves for (**A**) progression-free survival (PFS) and (**B**) overall survival (OS) in patients with irAEs or not. A statistically significant OS and PFS difference were noted among those experiencing any irAEs versus those who did not (*P* < 0.05).

The Kaplan-Meier survival analysis was performed to evaluate the impact of irAEs on progression-free survival (PFS) and overall survival (OS) in patients treated with PD-1 inhibitors. Compared with no irAEs, the development of irAEs was significantly associated with increased PFS (median 7.1 months; 95% CI, 1.9-12.3 versus 2.8 months; 95% CI, 2.7-2.9; *P* = 0.007) and OS (median 20.5 months; 95% CI, 15.2-25.8 versus 8.0 months; 95% CI, 6.7-9.3; *P* = 0.002) (Figure 1A and 1B).

**Figure 2:**
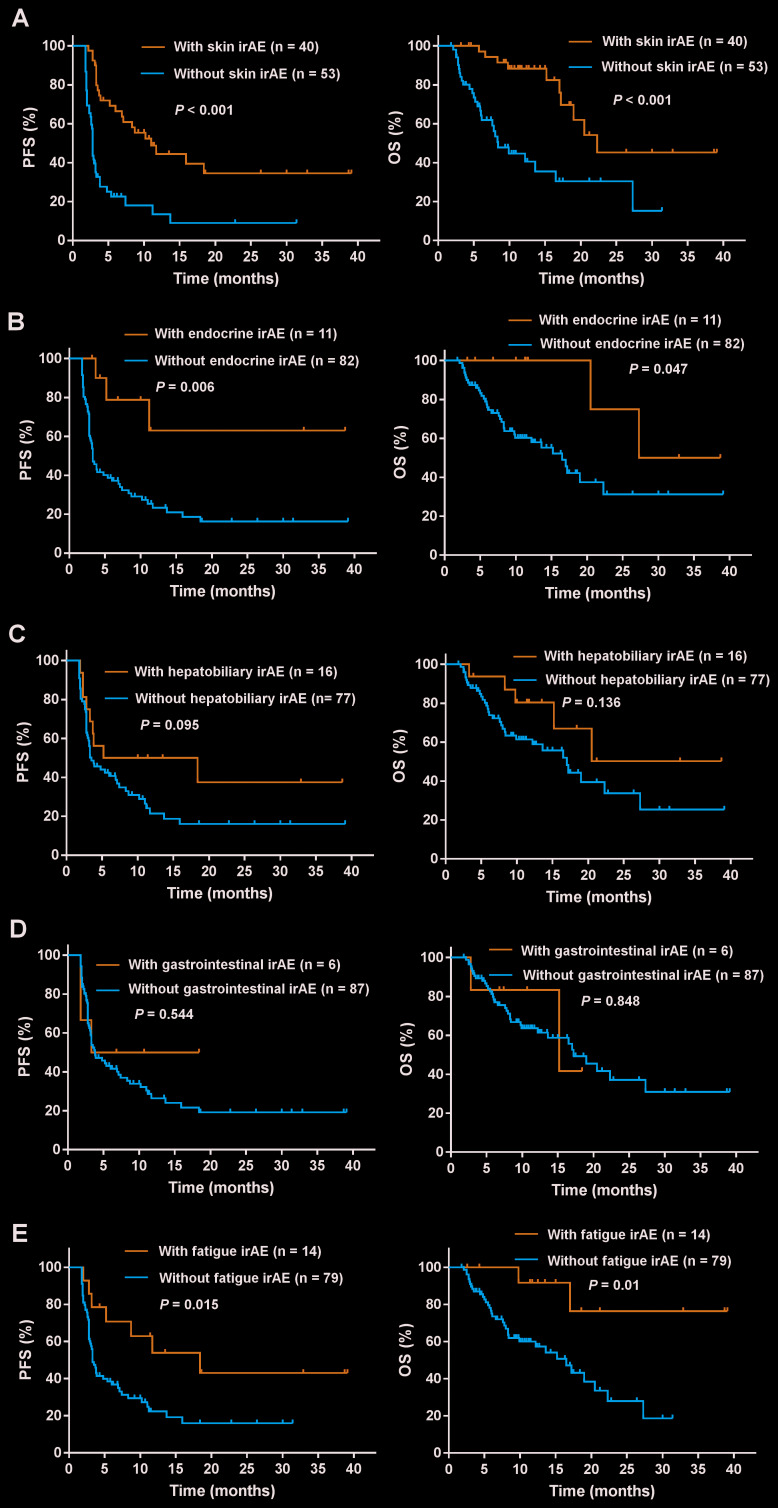
**Subgroup analysis to evaluate the association between common irAEs and survival.** Shown are the PFS and OS curves for patients with or without (**A**) skin irAEs, (**B**) endocrine irAEs, (**C**) hepatobiliary irAEs, (**D**) gastrointestinal irAEs, and (**E**) fatigue. The PFS and OS were significantly associated with skin, endocrine, and fatigue irAEs (*P* < 0.05).

**Figure 3:**
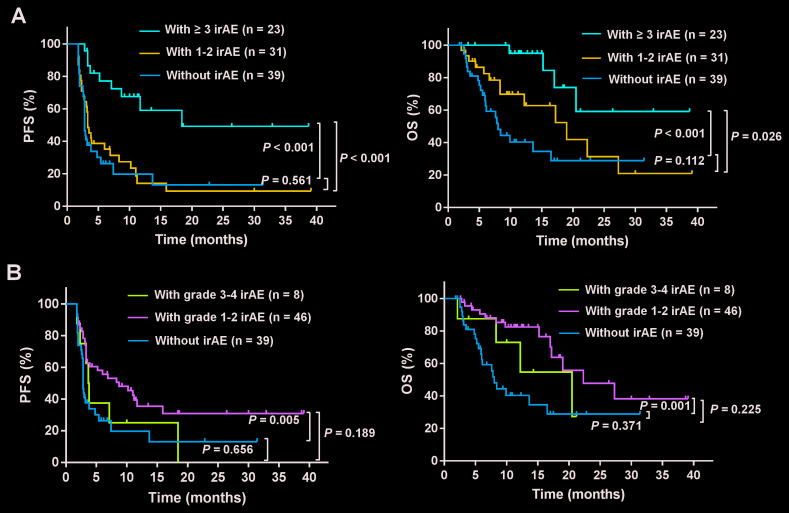
**Subgroup analysis to evaluate the association between the number or grade of the irAEs and the prognosis.** Shown are the PFS and OS curves for patients with irAEs of different (**A**) numbers and (**B**) grades. Patients with three or more irAEs showed longer PFS and OS when compared with those with one to two irAEs or none (*P* < 0.05). Patients with grade 1 to 2 irAEs showed longer PFS and OS when compared with those with no irAEs (*P* < 0.01).

The analysis of the association between clinical outcomes and common irAEs revealed that increased PFS was significantly associated with skin irAEs (median 11.0 months; 95% CI, 6.5-15.5 versus 2.8 months; 95% CI, 2.7-2.9, *P* < 0.001), endocrine irAEs (median Not reached (NR); 95% CI, NR-NR versus 3.3 months; 95% CI, 2.7-3.9, *P* = 0.006), and fatigue irAEs (median 18.4 months; 95% CI, 4.1-32.7 versus 3.3 months; 95% CI, 2.8-3.8, *P* = 0.015, respectively). Similarly, increased OS was also significantly associated with skin irAEs (median 22.3 months; 95% CI, NR-NR versus 8.4 months; 95% CI, 5.6-11.2, *P* < 0.001), endocrine irAEs (median 27.3 months; 95% CI, NR-NR versus 16.5 months; 95% CI, 12.7-20.3, *P* = 0.047) and fatigue (median NR; 95% CI, NR-NR versus 16.5 months; 95% CI, 13.3-21.7, *P* = 0.01) (Figure 2A, 2B, and 2E). In contrast, no differences in PFS and OS were observed between patients with and without hepatobiliary and gastrointestinal irAEs (Figure 2C and 2D). Additionally, we also assessed the association between the numbers and grades of irAEs and the prognosis in patients. Patients with three or more irAEs when compared with those with none showed a longer PFS (median 18.4 months; 95% CI, NR-NR versus 2.8 months; 95% CI, 2.7-2.9, *P* < 0.001) and OS (median NR; 95% CI, NR-NR versus 8.0 months; 95% CI, 6.7-9.3, *P* < 0.001). Similarly, patients with three or more irAEs when compared with those with one to two irAEs also showed longer PFS (median 18.4 months; 95% CI, NR-NR versus 3.3 months; 95% CI, 2.6-4.0, *P* < 0.001) and OS (NR; 95% CI, NR-NR versus 19.0 months; 95% CI, 10.1-27.9, *P* = 0.026) (Figure 3A). However, there were no statistically significant differences in the PFS and OS in patients with one to two irAEs when compared with those with no irAEs (Figure 3A). In addition, patients with grade 1 to 2 irAEs when compared with those with no irAEs showed longer PFS (median 8.7 months; 95% CI, 3.1-14.3 versus 2.8 months; 95% CI, 2.7-2.9, *P* = 0.005) and OS (22.3 months; 95% CI, 10.6-34.0 versus 8.0 months; 95% CI, 6.7-9.3 *P* = 0.001) (Figure 3B). There were no significant differences in the PFS and OS of patients with grade 3 to 4 irAEs when compared with those with grade 1 to 2 or no irAEs (Figure 3B). Moreover, the effects of irAEs on the prognosis of patients treated with PD-1 inhibitors were further evaluated by the Cox proportional hazards regression analyses. Multivariable analysis revealed that any irAE and the number of irAEs were significantly associated with increased PFS. Increased OS was also significantly associated with any irAE and the number of irAEs (Table 4).

**Table 4:** Cox proportional hazard regression analysis of the effect of immune-related adverse events development on progression-free survival and overall survival.

**Survival**	**Univariate analysis**			**Multivariate analysis^b^**		
	**HR**	**95% CI**	***P***	**HR**	**95% CI**	***P***
**PFS**						
Any irAEs	0.509	0.305-0.849	0.01^a^	0.521	0.309-0.877	0.014^a^
Skin irAEs	0.298	0.174-0.510	<0.001^a^	0.297	0.172-0.513	<0.001^a^
Endocrine irAEs	0.232	0.073-0.742	0.014^a^	0.269	0.083-0.874	0.029^a^
Hepatobiliary irAEs	0.688	0.375-1.263	0.228			
Gastrointestinal irAEs	0.939	0.347-2.544	0.902			
Fatigue irAEs	0.390	0.176-0.899	0.021^a^	0.410	0.181-0.927	0.032^a^
IrAEs number	0.532	0.384-0.736	<0.001^a^	0.547	0.389-0.769	0.001^a^
irAEs grade	0.678	0.438-1.049	0.081			
**OS**						
Any irAEs	0.366	0.191-0.699	0.002^a^	0.462	0.235-0.909	0.025^a^
Skin irAEs	0.257	0.124-0.535	<0.001^a^	0.288	0.132-0.628	0.002^a^
Endocrine irAEs	0.258	0.061-1.087	0.065			
Hepatobiliary irAEs	0.497	0.195-1.264	0.142			
Gastrointestinal irAEs	0.806	0.217-2.991	0.747			
Fatigue irAEs	0.186	0.044-0.779	0.021^a^	0.252	0.085-1.092	0.065
IrAEs number	0.450	0.288-0.705	<0.001^a^	0.529	0.326-0.857	0.01^a^
IrAEs grade	0.514	0.293-0.903	0.021^a^	0.641	0.357-1.151	0.136

Abbreviations: irAEs, immune-related adverse events; PFS, progression-free survival; OS, overall survival; HR, hazard ratio, CI, confidence interval

^a^ *P* value < 0.05

^b^ Covariables included metastasis stage (yes versus no), LDH levels (≤UNL versus>UNL), and liver metastases (yes versus no).

## DISCUSSION

In this study, we retrospectively analyzed the irAE profiles of PD-1 inhibitors including pembrolizumab and nivolumab. Using a relatively large sample size, we demonstrate that irAEs occurring before disease progression are associated with clinical outcomes in Chinese patients with advanced melanoma. Among the 93 patients, we found that 58.1% of them experienced irAEs with a potential immunological etiology. Most of the irAEs were typically mild to moderate in intensity, and only 8.6% of the patients experienced severe irAEs (grade 3 to 4). Consistent with previous reports [14, 28], the most commonly observed irAEs were rash, pruritus, vitiligo, thyroiditis, hepatitis, fatigue, and pyrexia. However, the incidence rates of certain types of irAEs were slightly different from those previously reported. In our study, after the commencement of treatment with PD-1 inhibitors, irAEs such as pruritus, rash, vitiligo, and hepatitis occurred in 30.1%, 24.7%, 16.1% and 8.6% of patients, respectively. These proportions are marginally higher than those reported in previous clinical trials (14.1-21%, 12-15%, 6-10.7%, and 0-1.1%, respectively) [4, 34–36]. However, the incidence rates of diarrhea and fatigue (5.4% and 15.1 %, respectively) in our study were lower than those previously reported (8-16.9% and 19.9-25%, respectively) [4, 34–36]. One reason for this discrepancy is the different ethnicity, and thereby leads to differences in the baseline characteristics of patients in our study compared with those in other studies. Compared with our study, there were relatively fewer Chinese patients enrolled in the KEYNOTE-001, KEYNOTE-002, KEYNOTE-006, CheckMate-037, and CheckMate-066 trials, which could account for the differences in the irAE profiles.

It has been proposed that the development of irAEs is associated with response to ICIs. This phenomenon was first described in melanoma patients who received ipilimumab therapy [24]. More recently, a retrospective analysis of 576 patients with advanced melanoma received nivolumab monotherapy in United States found a significantly higher ORR in patients who experienced irAEs of any grade than those who did not, with greater benefit in patients with three or more or one to two treatment-related AEs than those with none [27]. Consistent with these findings, our study also demonstrated that the ORR and DCR were significantly better in patients who experienced irAEs of any grade than those who did not. However, we found stronger associations between clinical outcomes and those with three or more irAEs than those with less than three irAEs. We also found that while the ORR and DCR were significantly higher in patients with grade 1 to 2 irAEs than those with no irAEs, but no significantly better outcomes were observed in patients with grade 3 to 4 irAEs than those with no irAEs. Furthermore, we found that patients with grade 3 to 4 irAEs showed poorer clinical outcomes when compared with those with grade 1 to 2 irAEs. A recent retrospective study also found that while ORR was significantly associated with low-grade irAEs in non-melanoma patients treated with PD-1 inhibitors, no significant relationship was found with high-grade irAEs [37]. The poorer clinical response in patients who developed grade 3 to 4 irAEs is potentially due to the termination of immunotherapy when faced with irAEs and the use of steroids.

Prior studies have demonstrated an association between the development of irAEs and clinical survival in tumor patients treated with PD-1 inhibitors. Analysis of 148 melanoma patients treated with nivolumab in the United States, the OS was greater in patients with irAEs especially three or more than those who had no irAEs [25]. A previous prospective study also reported that early irAEs are associated with a better PFS in NSCLC patients receiving nivolumab treatment [18]. Another retrospective study analyzed NSCLC patients treated with pembrolizumab from a single center in the KEYNOTE-001 trial and found that the patients who experienced irAEs had increased PFS and OS, compared to those who did not [20]. Consistent with these previous studies, we also found that the development of irAEs was significantly associated with increased PFS and OS, particularly in patients with three or more irAEs. In addition, we found that prolonged survival of advanced melanoma patients treated with PD-1 inhibitors was associated with skin and endocrine irAEs, as well as fatigue, but not with hepatobiliary and gastrointestinal irAEs. Our findings are consistent with some other studies that have showed an association between skin irAEs and prolonged survival in patients with NSCLC and melanoma treated with nivolumab [25, 28]. Moreover, a previous study on malignant melanoma revealed clear survival benefits in patients who received immunotherapy and developed vitiligo-like depigmentation [26]. In contrast to our results, it has been reported that hypothyroidism or hyperthyroidism was not associated with prolonged survival in malignant melanoma [25]. However, thyroid dysfunction was associated with improved OS of NSCLC patients treated with pembrolizumab [12].

The mechanism underlying the association of irAEs with the efficacy and clinical outcomes of treatment with PD-1 inhibitors remains unclear. However, previous studies have demonstrated that melanocytes and melanomas shared common antigens (e.g., MART-1, gp100, and tyrosinase related proteins 1 and 2) and lymphocytes directed against the tumor could cross-react with normal melanocytes and cause irAEs in the skin [11, 13, 28, 38]. In view of these findings, antigen sharing was considered the most likely cause of this association. Therefore, we speculate that irAEs can predict a strong activation of the immune system following the inhibition of PD-1, which can explain the better prognosis in melanoma patients, give the sensitivity of these tumors to immunotherapy.

In conclusion, this retrospective study demonstrated that irAEs were associated with the efficacy of PD-1 inhibitors in Chinese patients with advanced melanoma. Moreover, we showed that patients with at least three irAEs of grade 1 or 2, which include dermal, endocrine and fatigue were most likely to benefit from the inhibition of PD-1. However, our findings should be validated prospectively in subsequent analyses in larger cohorts of patients with advanced melanoma.

## MATERIALS AND METHODS

### Patient population

We performed a retrospective study of Chinese patients with advanced melanoma who received PD-1 inhibitor monotherapy at the Sun Yat-sen University Cancer Center between August 2014 and March 2018. Patients with histologically confirmed advanced melanoma derived from skin and non-skin sections were included in this study. The included patients were treated with intravenous pembrolizumab (2 mg/kg every 3 weeks) or nivolumab (3mg/kg every 2 weeks). After excluding 11 patients who received only one dose of the drug, and 2 patients who died before the first imaging evaluation, a total of 93 patients were finally enrolled in our study. The electronic medical records were reviewed to obtain information including patient demographic, ECOG status, primary site, metastasis stage, BRAF V600E status, prior therapy, number of anti-PD-1 drug doses received, any irAEs, use of corticosteroids, findings of the imaging evaluation, date of progression and start of new treatment or death. The follow-up was ended on June 1, 2018. The Institutional Review Board of the Sun Yat-sen University Cancer Center approved the study, and all the patients provided written informed consent.

### Assessments

IrAEs are defined as AEs with a potential immunologic cause that require frequent monitoring and intervention with immunosuppressive and/or endocrine therapy [5, 39–44]. All irAEs were graded according to the National Cancer Institute Common Terminology Criteria for Adverse Events (version 4.0). Radiological evaluations (CT or MRI) were performed at baseline and subsequently at every 8 to 12 weeks to assess tumor responses. Tumor assessments were made based on the Response Evaluation Criteria in Solid Tumors (RECIST version 1.1), and included complete remission (CR), partial remission (PR), stable disease (SD), and progressive disease (PD). The ORR (CR + PR) and DCR (CR + PR + SD) were also calculated. PFS was defined as the interval between the start of the treatment and disease progression or death due to any cause. OS was defined as the interval between the start of the treatment and death due to any cause. Patients who did not progress or were still alive at the last follow-up date were censored.

### Statistical analysis

Clinical and demographic characteristics of the patient with and without irAEs were compared using the Fisher exact test for categorical variables. ORRs and DCRs with 95% CIs were estimated using the Clopper-Pearson method. The Fisher exact tests were used to determine the associations between the number/grade of irAEs and the tumor response rates including ORRs and DCRs. The differences in the PFS and OS curves (estimated by the Kaplan-Meier method) based on the absence or presence of any irAEs which were observed before disease progression was evaluated using the log-rank test. We used a Cox proportional hazards model to calculate HRs and 95% CIs. A multivariable analysis was performed with adjustment for metastasis stage, LDH levels, and liver metastases. A two-tailed p value < 0.05 was considered to be statistically significant. All the statistical analyses were performed using the IBM SPSS 19.0 software.
